# The chemodiversity of paddy soil dissolved organic matter correlates with microbial community at continental scales

**DOI:** 10.1186/s40168-018-0561-x

**Published:** 2018-10-19

**Authors:** Hong-Yi Li, Hang Wang, Hai-Tiao Wang, Pei-Yong Xin, Xin-Hua Xu, Yun Ma, Wei-Ping Liu, Chang-Yun Teng, Cheng-Liang Jiang, Li-Ping Lou, Wyatt Arnold, Lauren Cralle, Yong-Guan Zhu, Jin-Fang Chu, Jack A Gilbert, Zhi-Jian Zhang

**Affiliations:** 1 College of Environment and Natural Resource Sciences, Zhejiang University, 866 Yuhangtang Ave, Hangzhou, 310058 China; 2 National Plateau Wetlands Research Center, Southwest Forestry University, 300 Bailongsi, Kunming, 650224 China; 3 The Microbiome Center, Biosciences Division, Argonne National Laboratory, Lemont, IL 60439 USA; 4 Department of Surgery, University of Chicago, 5640 South Ellis Avenue, Chicago, IL 60637 USA; 5 National Center of Plant Gene Research (Beijing), Institute of Genetics and Developmental Biology, Chinese Academy of Sciences, West Beichen Road, Chaoyang District, Beijing, 100101 China; 6 College of Biological and Environmental Engineering, Zhejiang University of Technology, 18 Chaowang Ave, Hangzhou, 310014 China; 7 Hangzhou Gusheng Agricultural Technology Company Limited, Chongxian Innovation Industrial Park, Chongxian Ave, Hangzhou, 311108 China; 8 Key Lab of Urban Environment and Health, Institute of Urban Environment, Chinese Academy of Sciences, 1799 Jimei Ave, Xiamen, 361021 China; 9 China Academy of West Region Development, Zhejiang University, 866 Yuhangtang Ave, Hangzhou, 310058 China

**Keywords:** Dissolved organic matter, Paddy soil, Chemodiversity, Microbial diversity, FT-ICR-MS

## Abstract

**Background:**

Paddy soil dissolved organic matter (DOM) represents a major hotspot for soil biogeochemistry, yet we know little about its chemodiversity let alone the microbial community that shapes it. Here, we leveraged ultrahigh-resolution mass spectrometry, amplicon, and metagenomic sequencing to characterize the molecular distribution of DOM and the taxonomic and functional microbial diversity in paddy soils across China. We hypothesized that variances in microbial community significantly associate with changes in soil DOM molecular composition.

**Results:**

We report that both microbial and DOM profiles revealed geographic patterns that were associated with variation in mean monthly precipitation, mean annual temperature, and pH. DOM molecular diversity was significantly correlated with microbial taxonomic diversity. An increase in DOM molecules categorized as peptides, carbohydrates, and unsaturated aliphatics, and a decrease in those belonging to polyphenolics and polycyclic aromatics, significantly correlated with proportional changes in some of the microbial taxa, such as *Syntrophobacterales*, *Thermoleophilia*, *Geobacter*, *Spirochaeta*, *Gaiella*, and *Defluviicoccus*. DOM composition was also associated with the relative abundances of the microbial metabolic pathways, such as anaerobic carbon fixation, glycolysis, lignolysis, fermentation, and methanogenesis.

**Conclusions:**

Our study demonstrates the continental-scale distribution of DOM is significantly correlated with the taxonomic profile and metabolic potential of the rice paddy microbiome. Abiotic factors that have a distinct effect on community structure can also influence the chemodiversity of DOM and vice versa. Deciphering these associations and the underlying mechanisms can precipitate understanding of the complex ecology of paddy soils, as well as help assess the effects of human activities on biogeochemistry and greenhouse gas emissions in paddy soils.

**Electronic supplementary material:**

The online version of this article (10.1186/s40168-018-0561-x) contains supplementary material, which is available to authorized users.

## Background

Paddy fields, 90% of which are in Asia, feed more than half of the world’s population [1]. The continuous flooding in bunded fields of cultivated rice (*Oryza sativa*) utilizes 24–30% of the world’s developed freshwater resources and represents one of the major sources of inland aquatic dissolved organic matter (DOM) [1, 2]. High concentrations and fluxes of DOM from plant debris during flooding seasons trigger microbial activity, while anaerobic conditions stabilize DOM against microbial decay via interactions with clay minerals and iron oxides [1, 3]. DOM plays a central role in biogeochemical processes in both flooded and unflooded paddy soils, as well as an active role in the global carbon cycle [1, 4].

Recently, the evidence-based soil continuum model questioned the secondary synthesis of “humic substances,” or the “humification,” and interpreted organic debris as a unique source of soil organic matter (SOM) and DOM [5]. This theory emphasized the inherent association between soil microbial metabolism and DOM heterogeneity. Microbes play an important role in carbon and nitrogen cycling as well as methane production and consumption in soils [1, 6, 7], which influences carbon balance, greenhouse gas production, crop productivity, and water eutrophication [8]. Therefore, a growing body of research has focused on the biogeography of microbial communities [9–11]. However, despite efforts to characterize the drivers of DOM concentration dynamics [8, 12–15], no attempts have been made to combine these data so as to understand the associated properties of each and the environmental factors that drive them. Ultrahigh-resolution Fourier transform ion cyclotron resonance mass spectrometry (FT-ICR-MS) enables detailed characterization of DOM molecular distribution [13, 16, 17]. This approach has been applied to marine [18, 19] and inland water [4, 20, 21], and a handful of comparative experiments have examined soil DOM at the molecular level [3, 14, 15, 22]; however, the microbial taxonomic and metabolic structures that influence the molecular distribution of soil DOM remain unknown.

To our knowledge, no comprehensive study has yet been performed to elucidate the natural relationship between microbial metabolisms and DOM molecular distribution in paddy soils on continental scales. We apply FT-ICR-MS plus amplicon and metagenomic sequencing to characterize the association between microbial community structure and function with DOM molecular composition in flooded paddy soils. We hypothesized that taxonomic and functional composition of soil microbial communities is significantly associated with DOM molecular composition in paddy soils and, moreover, that geographic and edaphic factors significantly affect this interdependence.

## Results

### Microbial and DOM biogeography in paddy soils

**Fig. 1:**
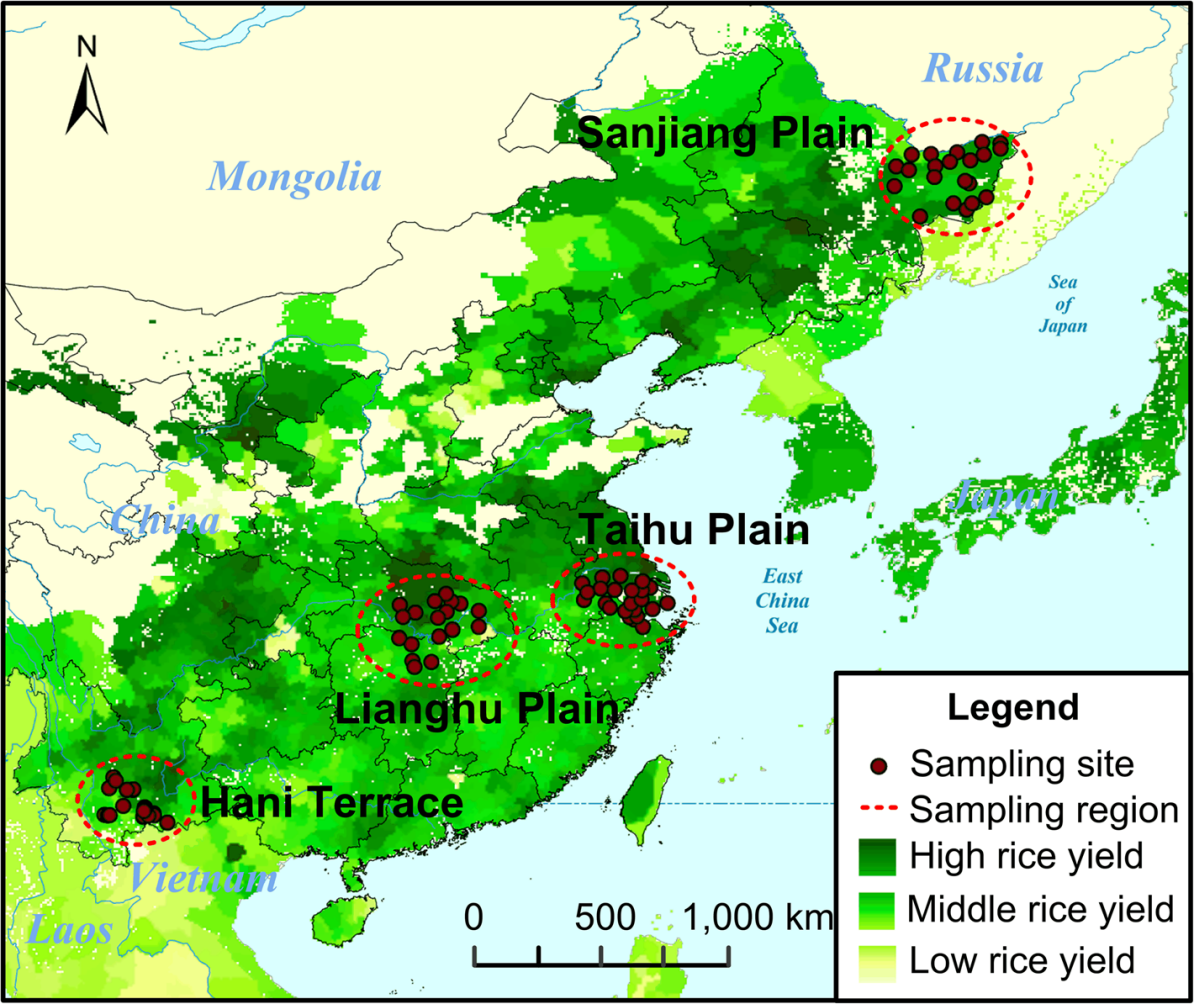
Map of sampling sites (dark-red dots) across four typical Chinese rice-growing regions (red dotted ellipses). It shows broad patterns of rice field. The map was colored to depict circa the year 2000 area (harvested) and yield of rice crops of China [90]

**Fig. 2:**
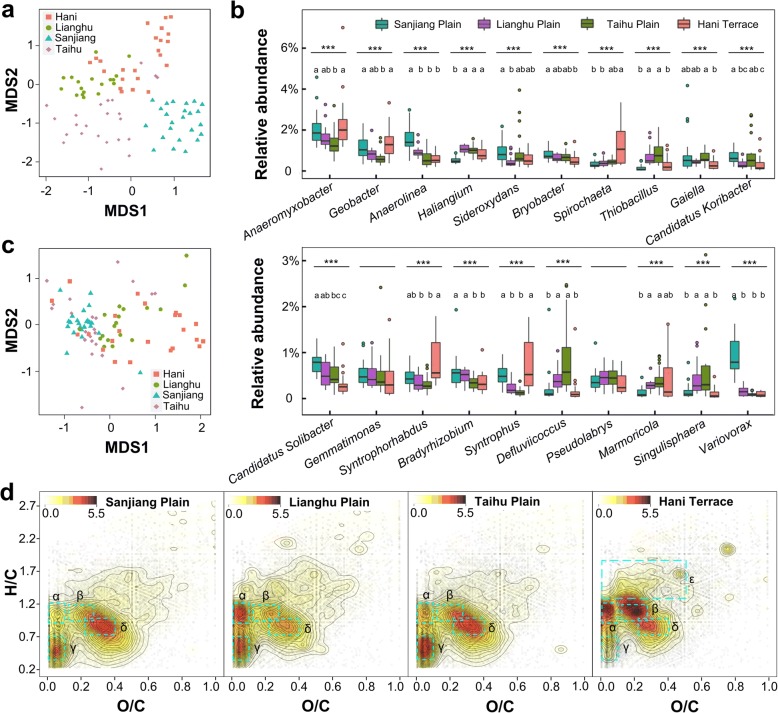
Distributions of DOM molecules and bacteria across 88 paddy soils from four regions. **a** Non-metric multidimensional scaling of bacterial OTUs (matrix: variance adjusted weighted UniFrac, stress = 0.1918). **b** Boxplots showing regional differences of top 20 genera. Genera with significant differences (Kruskal-Wallis test) were labeled with "*" (*P* < 0.05), "**" (*P* < 0.01), and "***" (*P* < 0.001). For each genus, paired boxes containing no same letter are considered to be significantly different (*P* < 0.05, Dunn’s test). **c** Non-metric multidimensional scaling of DOM molecules (matrix: Bray-Curtis, stress = 0.1479). **d** Kernel density plots of DOM formula in van Krevelen diagrams. Darker color indicates a higher molecular density. Accumulated areas α, β, ɣ, δ, and ε are considered to be clusters of highly unsaturated/aromatic hydrocarbon, phenolics, polycyclic aromatics, polyphenols, and unsaturated aliphatics or peptides, respectively

Across four rice-growing regions in China, we collected soil samples from 88 flooded paddy fields, wherein most rice plants were at the tillering phase (Fig. 1 and Table 1). Based on 16S rRNA gene sequencing, *Anaeromyxobacter* (1.9% ± 0.9%), *Geobacter* (1.0% ± 0.5%), *Anaerolinea* (0.9% ± 0.6%), and *Haliangium* (0.8% ± 0.3%) were the most abundant genera. Bacterial richness (Chao1 and observed species) and diversity (Shannon and PD whole tree) were significantly different between regions (Additional file 1: Figure S1A), with the lowest diversity and richness observed in samples from Sanjiang Plain (*P* < 0.05, Dunn’s test). Microbial *β*-diversity (variance adjusted weighted UniFrac; VAW-UniFrac) was significantly correlated to the distance between sites (Mantel *r* = 0.52, *P* < 0.001; Fig. 2a). The proportions of the dominant taxa at each phylogenetic level differed significantly by region (Additional file 1: Figure S2). Among the 20 most abundant genera, only *Gemmatimonas* and *Pseudolabrys* were stably abundant across regions, while the other genera were significantly different between regions (Fig. 2b).

**Table 1:** Various characteristics of the four typical Chinese rice-growing regions

	Hani Terrace	Taihu Plain	Sanjiang Plain	Lianghu Plain
Administrative region	Yunnan Province.	Jiangsu, Zhejiang, Anhui Provinces, and Shanghai Municipality	Heilongjiang Province	Hunan and Hubei Provinces
Longitude range	100°55′27″–103°14′57″	118°38′12″–121°47′48″	130°12′45″–134°8′45″	111°50′51″–114°48′12″
Latitude range	22°46′44″–24°28′13″	30°3′19″–31°56′15″	45°16′53″–48°2′40″	28°34′7″–31°16′21″
Climate	3-D climate, (sub)tropics	subtropics	temperate	subtropics
Elevation (m)	350–3000	0–45	50–160	10–100
Rice cultivation history	~ 1200 years	~ 5000 years	~ 60 years	~ 4000 years
Rice cultivars (*Oryza sativa L*.) [89]	Most are *japonica*	Half are *indica* and half are *japonica*	All are *japonica*	Most are *indica*
Cultivation method	traditional	modern and small-scale	modern and large-scale	modern and medium-scale
Mean pH	6.07 ± 0.60	6.60 ± 0.70	6.32 ± 0.32	6.80 ± 0.68
Mean annual temperature (°C)	16.21 ± 1.77	15.66 ± 0.41	2.85 ± 0.78	16.68 ± 0.35
Mean annual precipitation (mm/d)	3.20 ± 0.19	3.74 ± 0.19	1.86 ± 0.13	3.45 ± 0.29
Dissolved organic carbon (mg/ml)	23.51 ± 6.84	31.68 ± 11.41	29.28 ± 8.39	31.61 ± 14.40

DOM analysis revealed 81,759 compounds in total with an average of 8262 ± 1187 compounds at each site (Additional file 2: Table S1). A core group of 18,538 molecules was observed in at least 10 sites; 12,791 of these compounds could be assigned putative molecular formulae. Chao1 of DOM molecules was significantly different between the four regions (Additional file 1: Figure S1B), while Observed species and Shannon diversity were not. *β*-diversity (Bray-Curtis) of DOM was still significantly associated with distance between sites (Mantel *r* = 0.24, *P* < 0.001; Fig. 2c). Weighted density plots of DOM components in van Krevelen diagrams (Fig. 2d) visualized the significant influence of geographic region on the abundance of DOM molecular groups (permutational multivariate analysis of variance, PERMANOVA *r*^2^ = 0.17, *P* < 0.001). For example, unsaturated/aromatic hydrocarbons were enriched in Hani Terrace and Lianghu Plain samples (area α), phenolics were enriched in the Hani Terrace samples (area β), polycyclic aromatics were enriched in Taihu Plain samples (area ɣ), and polyphenols were enriched in Sanjiang Plain and Taihu Plain samples (area δ). Unsaturated aliphatics and peptides were enriched in samples of Hani Terrace (area ε).

**Fig. 3:**
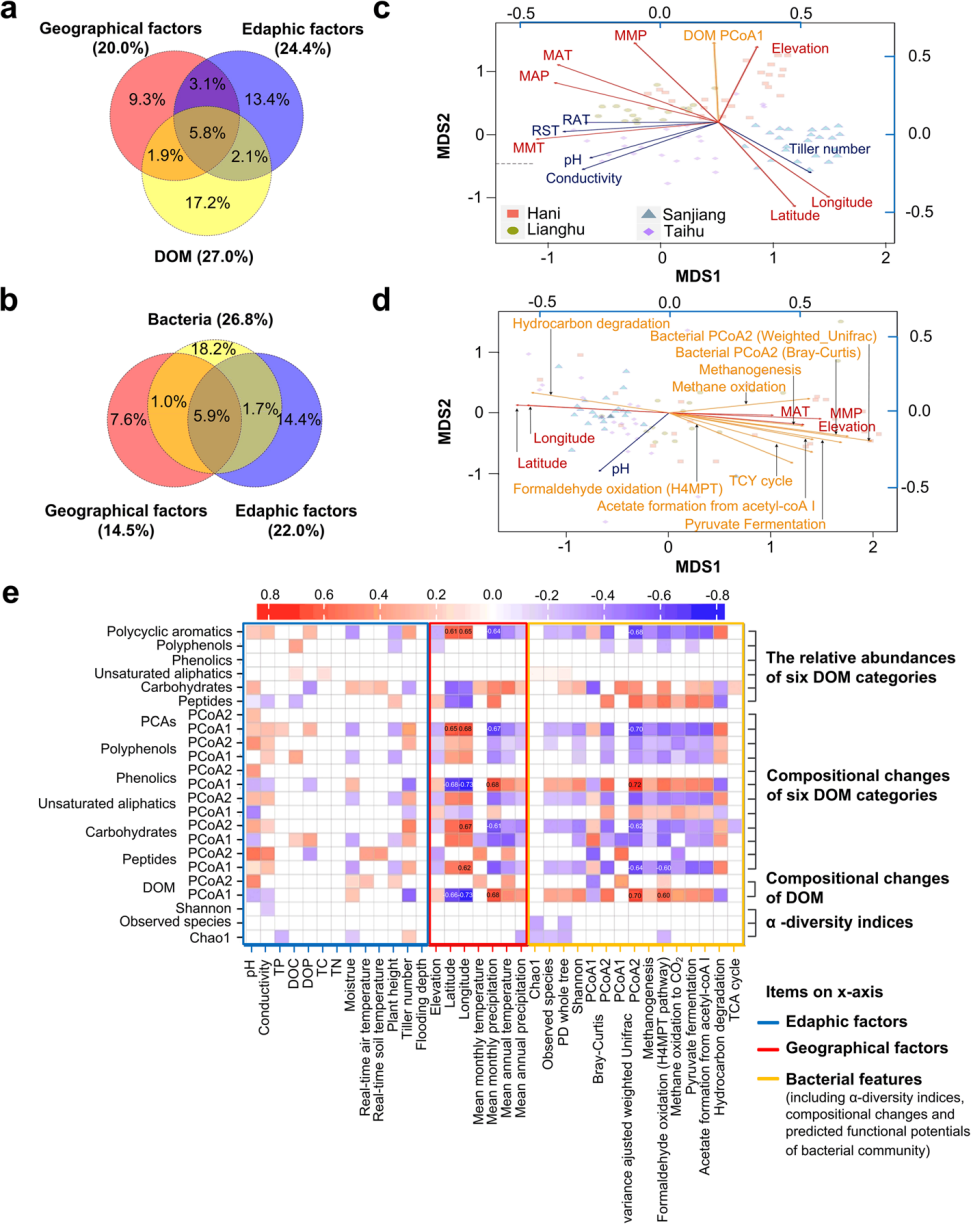
Associations between DOM composition, bacterial community, and the environmental drivers. **a** The influences of geographical factors, edaphic factors, and DOM composition features on the bacterial community structure estimated via canonical correspondence analyses (CCA). **b** The influences of geographical factors, edaphic factors, and bacteria community features on the DOM composition estimated via CCA. The percentages represent the variance explained. **c**, **d** Multivariate analysis of microbial or molecular data and drivers using non-metric multidimensional scaling (NMDS). Ordinations are based on Bray-Curtis (**c**, stress = 0.1656; **d**, stress = 0.1479). Geographical factors, edaphic factors, and DOM composition (**c**) or bacteria (**d**) community were fit to the ordination using *envfit* function, respectively. Only factors with significance level ≤ 0.001 were shown. **e** Spearman’s rank correlations between DOM diversity features (*y* axis) and edaphic, geographical, and bacterial factors (*x* axis), with color coded in blue, red, and yellow, respectively. All factors imported and their influences are listed in Additional file 3: Table S2 and Additional file 4: Table S3. MAP, mean annual precipitation; MAT, mean annual temperature; MMP, mean monthly precipitation; MMT, mean annual temperature; RST, real-time soil temperature; RAT, real-time air temperature

Canonical correspondence analysis (CCA) and partial CCA were performed to estimate the contribution of environmental factors to the variance in microbial and DOM diversity across sites. The variance in microbial community was best explained by the variance in DOM composition (27%; Fig. 3a and Additional file 3: Table S2), with the converse being true as well (26.8%; Fig. 3b and Additional file 4: Table S3). This was confirmed using Procrustes analysis, which demonstrated that the dissimilarity of DOM and microbial communities between samples was significantly and strongly correlated (*m12*^2^ = 0.66, *P*_Monte Carlo_ < 0.001). More specifically, the second principal coordinate (PCo2) of the microbial Bray-Curtis (5.5%) and VAW-UniFrac distances (5.9%) as well as functional potentials (3.0%–4.5%) were the main contributors of DOM variance; PCo1 of DOM Bray-Curtis distance (4.3%) was the main contributor of microbial variance, followed by DOM alpha diversity indicies and PCo1–2 of phenolics, peptides, polyphenols, and polycyclic aromatic (in the column of V.E. CCA and V.E. pCCA, Additional file 3: Table S2 and Additional file 4: Table S3). Individual edaphic factors of pH (3.5%), conductivity (3.7%), real-time air temperature (RAT; 3.5%), real-time soil temperature (RST; 4.6%), tiller number (4.3%), and all geographic factors (4.4–6.9%) also described microbial variance (Additional file 3: Table S2). It should be noted that conductivity was used to quantify water-soluble ions [23]; tiller number and plant height were used to roughly indicate the rice growth stage and the size of plants, and they were classified into edaphic factors for simplicity. Meanwhile, edaphic factors of pH (3.4%), dissolved organic carbon (DOC; 3.3%), and geographic factors of elevation (4.4%), latitude (4.3%), longitude (4.1%), and mean monthly precipitation (MMP; 4.3%) and mean annual temperature (MAT; 3.2%) also described the variance in DOM (Additional file 4: Table S3).

To confirm these findings, we fitted these factors to unconstrained non-metric multidimensional scaling (NMDS) ordination (Fig. 3c, d, Additional file 3: Table S2 and Additional file 4: Table S3). Significant correlations (*P* ≤ 0.001) were observed between the variances of microbial/DOM composition and MMP (*r*^*2*^ = 0.637/0.355), MAT (*r*^*2*^ = 0.742/0.200), pH (*r*^*2*^ = 0.407/0.409), and elevation (*r*^*2*^ = 0.342/0.347), as well as latitude (*r*^*2*^ = 0.613/0.358) and longitude (*r*^*2*^ = 0.553/0.305; Additional file 3: Table S2 and Additional file 4: Table S3). The associations between edaphic, geographical, bacterial (*x*-axis), and DOM (*y*-axis) factors were calculated using Spearman’s rank correlation (Fig. 3e). The PCo2 of the microbial Bray-Curtis and VAW-UniFrac distances, latitude, longitude, MMP, MAT, pH, and predicted functional potential were all strongly correlated with the PCo1 of DOM (*P* < 0.001; Fig. 3e and Additional file 4: Table S3). MMP, as well as the second coordinates (PCo2) of microbial Bray-Curtis and VAW-UniFrac distances, described the geographic variance in DOM (PCo1) and DOM features, while pH, and the PCo1 of microbial distances, described another pattern of DOM variance (PCo2) (Fig. 3e). Precipitation was positively correlated with the relative abundance of carbohydrates and peptides while negatively correlated with the relative abundance of polycyclic aromatic compounds (Fig. 3e and Additional file 1: Figure S3).

### Characterizing the association between DOM molecular and bacterial taxonomic composition

**Fig. 4:**
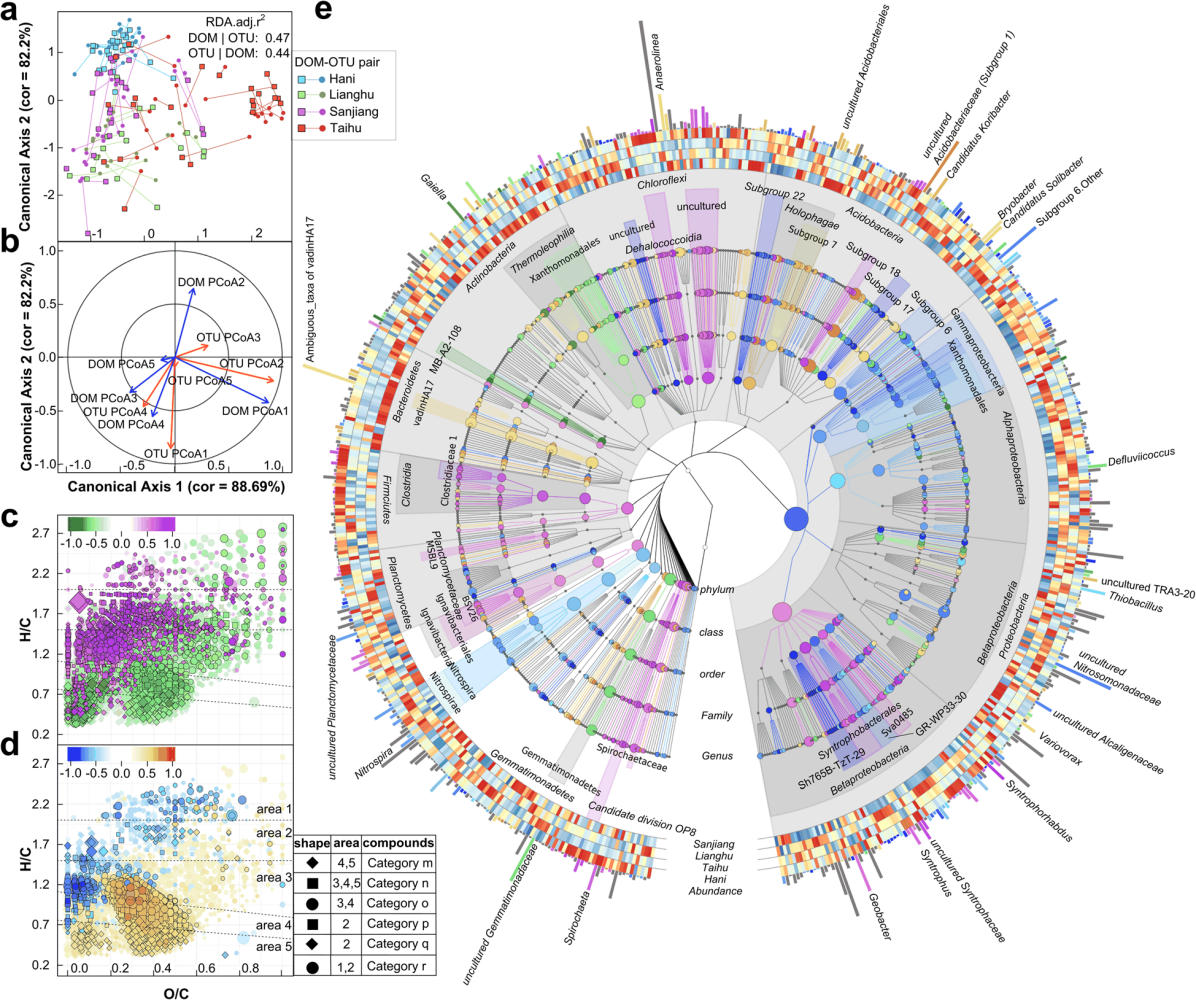
Associations between DOM composition and bacterial community. Canonical correlation analysis was conducted using first five principal coordinates (PCo1–5). **a** Combined score plots of the corresponding ordinations pairs of DOM composition and microbial community along the first and second canonical axes. The lengths of connecting lines represent the dissimilarities of DOM composition and microbial community. **b** Plot showing loading coefficients of the five pairs of principal coordinates imported on the corresponding first two pairs of canonical axes. **c**, **d** van Krevelen plots of DOM molecules showing positive and negative Spearman’s rank correlations with the DOM ordinations along the first (**c**) and second (**d**) canonical axes, indicating their association with microbial community dynamics. **e** Taxonomic cladogram showing positive and negative correlations with microbial ordinations along the first two canonical axes, indicating their association with DOM compositional changes. Rings of the cladogram provide a heatmap of the genera (> 0.01%) among the sampling regions with red and blue meaning more and less accumulated, respectively. Relative abundances of the taxa or genera are shown as the clade marker size or level 5 ring height. The color gradient bars in **c** and **d**, “purple to green” (for the first canonical axis) and “orange red to blue” (for the second canonical axis), indicate the values of coefficients. These color scales are also applied to the associations between taxa and microbial ordinations along the first and second canonical axes (**e**). Compounds category m: polycyclic aromatics; n: polyphenols and polycyclic aromatics with aliphatic chains; o: phenolic and highly unsaturated compounds; p: unsaturated aliphatics and aromatics with aliphatic chains; q: saturated fatty, sulfonic acids, and carbohydrates; r: N-containing compounds, i.e., peptides

We performed canonical correlation analysis (CCorA) to characterize the association between bacterial and DOM composition (Fig. 4a, b). Using the PCo1–5 axes of DOM and microbial Bray-Curtis matrices, we observed four out of five canonical axes with significant correlation coefficients (*P* < 0.01, chi-square test), which confirmed the multi-dimensional associations between bacterial community and DOM composition (*P* = 0.0001; Fig. 4a). Each pair of ordinations along canonical axis represents two correlated dimensions of the multivariate matrices of microbial community and DOM composition (e.g., Spearman’s rank correlation *ρ* = 0.89, *P* = 2.2 × 10^−16^ for the first pair; Fig. 4b).

DOM molecules and microbial taxa that were responsible for the first two pairs of correlated ordinations of DOM and microbial community were figured out using Spearman’s rank correlation test (Fig. 4c–e). Bacterial taxa showing significant correlation in the first canonical axis were enriched in Hani Terrace samples and attenuated in Sanjiang Plain samples. These included organisms (e.g., *Geobacter*, *Syntrophorhabdus*, and *Spirochaeta*) (Fig. 4e) that were positively correlated with highly unsaturated/aromatic hydrocarbon (Fig. 4c; area α in Fig. 2d) and phenolics (area β) and negatively correlated with polycyclic aromatics (area ɣ) and polyphenols (area δ). However, some taxa (e.g., *Gaiella* and *Defluviicoccus*) demonstrated an opposite trend (Fig. 4e). Also, other taxa and DOM compounds were correlated in the second canonical axis (Fig. 4d, e).

### Factors of the covariation between microbial taxa and DOM molecules

To determine the factors of covariation between the microbiome and DOM, we correlated MMP, MAT, and pH against the DOM and microbial community ordinations along the first two canonical axes using Spearman’s rank correlation test. For covariation along the first axis (Fig. 4b), MMP and pH showed strong and significant correlations (*ρ* = − 0.68/− 0.65, *P* = 3.85 × 10^−13^/9.03 × 10^−12^ for pH with DOM/microbial ordination; *ρ* = 0.52/0.46, *P* = 2.17 × 10^−7^/6.48 × 10^−6^ for MMP with DOM/microbial ordination), while MAT was not correlated (*P* > 0.1). For covariation along the second axis (Fig. 4b), MAT, MMP, and pH showed strong and significant correlations (*ρ* = − 0.40/− 0.59, *P* = 1.01 × 10^−4^/1.33 × 10^−9^ for pH with DOM/microbial ordination; *ρ* = − 0.51/− 0.50, *P* = 3.93 × 10^−7^/8.01 × 10^−7^ for MMP with DOM/microbial ordination; *ρ* = − 0.71/− 0.72, *P* = 9.50 × 10^−15^/2.30 × 10^−15^ for MAT with DOM/microbial ordination). In our study, since MMP and MAT changed in opposite trends along the increased elevation in Hani Terrace sites, MMP and MAT, as well as pH, were not correlated with each other (*P* > 0.05).

### DOM composition correlates with microbial functional potential

To characterize the microbial functional potential, four samples from each of the four regions were selected for deep shotgun metagenomic sequencing. The most abundant genera were *Streptomyces* (3.8%), *Anaeromyxobacter* (3.2%), *Bradyrhizobium* (3.0%), *Mycobacterium* (1.7%), *Solibacter* (1.3%), and *Geobacter* (1.2%). The abundance of this particular collection of taxa roughly paralleled what was previously observed in the 16S rRNA analysis (for details see Additional file 5 and Additional file 1: Figure S4).

**Fig. 5:**
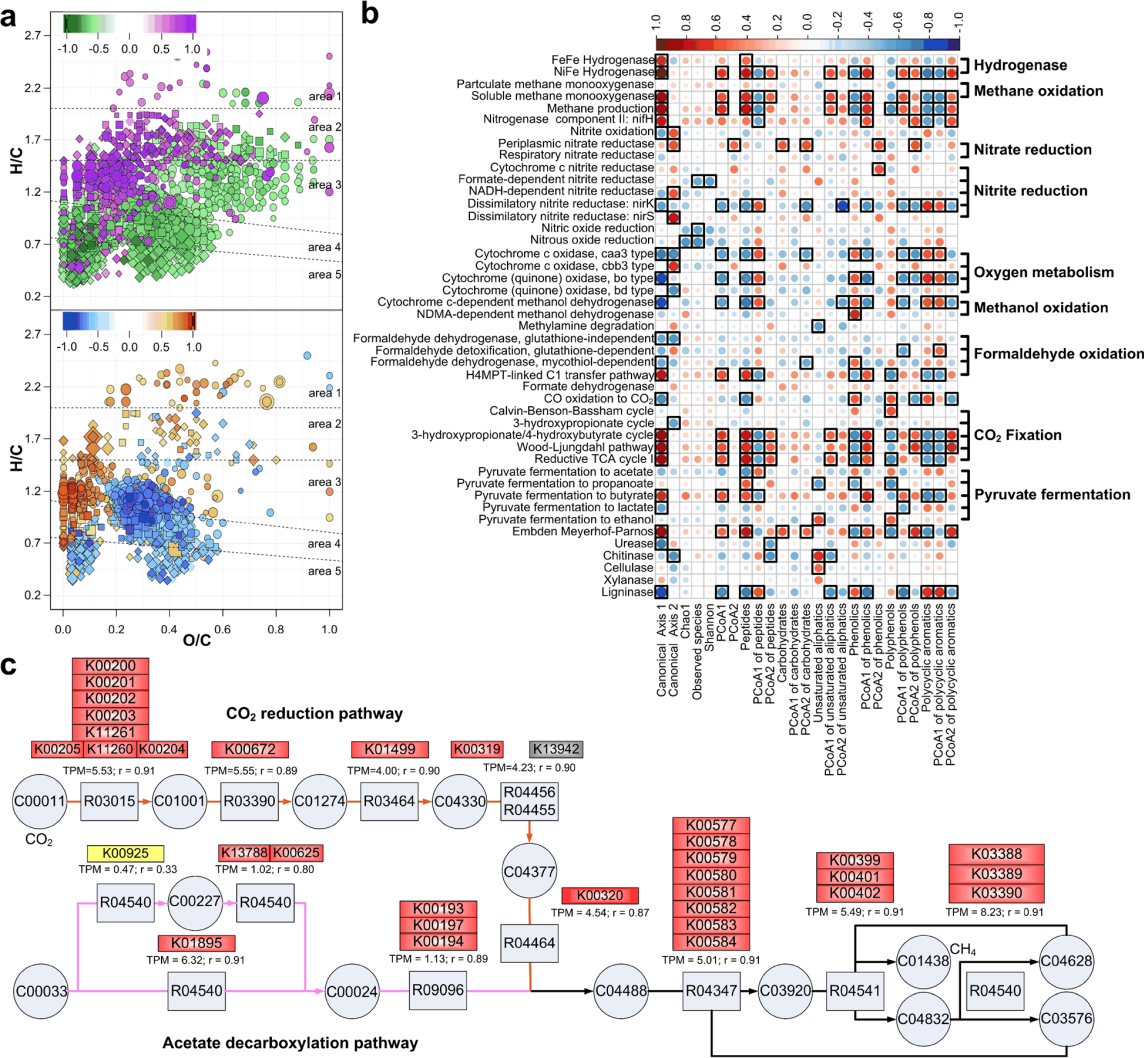
Associations between soil DOM composition and microbial functional capabilities estimated by metagenomics. **a** van Krevelen plot showing the positive and negative Spearman’s rank correlations of DOM molecules with the first canonical axis of canonical correlation analysis which indicates the correlation between DOM composition and FOAM orthologs. “Blue to red” gradient colors indicate the values of the coefficients. **b** Pearson’s correlation coefficient between relative abundances of biogeochemical functions (*y* axis) and DOM diversity features (*x* axis). Significant correlations (*P* < 0.05) are indicated by the black boxes. The first column of **b** shows the positive and negative Pearson’s correlation coefficients between biogeochemical functions and the first canonical axis. **c** FOAM orthologs correlated with DOM distributions, regarding methanogenesis in KEGG module M00567 and M00357. The protein catalog for quantification was restricted to methanogens (Additional file 9: Table S7) using Kaiju [70]. Moreover, it was further restricted to *Methanothrix* and *Methanosarcina* for aceticlastic methanogenesis. The red, blue, and yellow colors indicate significantly (*P* < 0.05) positive, negative, and nonsignificant (*P* > 0.05) Pearson’s correlation coefficients between FOAM orthologs and the DOM variance along the first canonical axis, respectively. Gray color indicates orthologs detected in less than eight samples, whereas white indicates orthologs undetected

The relationship between DOM composition and the relative abundance of metagenomic functional genes was determined by CCorA using PCo1–5 axes of DOM and PCo1–2 axes of FOAM (Functional Ontology Assignments for Metagenomes, a functional gene database) orthologs (explained 70% and 65% variances, respectively). We observed two canonical axes with significant correlation coefficients (*P* < 0.01, chi-square test), which confirmed the association between microbial function potential and DOM composition (*P* = 0.012, *r*_1_ = 0.89, *r*_2_ = 0.76). The association between DOM and FOAM orthologs (Fig. 5a) followed a similar trend to the association between DOM and microbial taxonomy (Fig. 4c, d). Redundancy analysis (built-in function of CCorA) indicated that the functional potential distribution could be predicted by the DOM distribution with an adjusted *R*^2^ of 0.70, while the distribution of DOM was correlated to functional potentials with an adjusted *R*^2^ of 0.35. Consistently, 109 out of 129 observed FOAM level 2 functional pathways were significantly (|*r*| > 0.5, *P* < 0.05) correlated with the variances of DOM in the first two canonical axes (Additional file 6: Table S4).

To avoid risk of false correlations, we further used marker genes of certain biogeochemical functions to qualitatively evaluate the associations (Fig. 5b). Consistently, functional genes involved in glycolysis (Embden-Meyerhof-Parnos, EMP), anaerobic C-fixation (light-independent), pyruvate fermentation (to butyrate), methanogenesis, methane oxidation (soluble methane monooxygenase), and hydrogen metabolism, as well as H4MPT-linked C1 transfer pathway (hydrogenotrophic methanogenesis associated), showed positive correlation with compositional changes in DOM along the first canonical axis, whereas those related to pyruvate fermentation (to lactate), carbon monoxide oxidation, urea degradation, nitrite oxidation, dissimilatory nitrite reduction (*nirK*), and ligninase, as well as the caa3-type and bo-type cytochrome oxidases, were negatively correlated (Fig. 5b). Compositional changes of DOM along the first canonical axis were characterized by increases in highly unsaturated/aromatic hydrocarbon and phenolics, peptides, and unsaturated aliphatics (O/C < 0.5), as well as decreases in polycyclic aromatics and polyphenols (Fig. 5a, upper panel). FOAM function categories associated with the tricarboxylic acid (TCA) cycle and homoacetogenesis were positively correlated with compositional change of DOM along the first canonical axis, whereas hydrocarbon degradation, cellular response to oxidative stress, and fatty acid oxidation pathways were negatively correlated (Additional file 6: Table S4).

To confirm the findings from this limited metagenomic dataset, Tax4fun [24] was used to predict the abundance of functional genes based on the 16S rRNA amplicon data. The correlation between predicted functional potential and DOM was mostly consistent with the correlation observed for the real metagenomic data (Additional file 5 and Additional file 7: Table S5). The metagenomic taxa that correlated with DOM along the first canonical axis of Fig. 4c also showed significant correlation with DOM along the first canonical axis of Fig. 5a. These include *Geobacter* (Pearson’s correlation coefficient, *r* = 0.83, *P* = 6.32 × 10^−5^), *Syntrophobacterales* (*r* = 0.94, *P* = 5.98 × 10^−8^), *Spirochaeta* (*r* = 0.92, *P* = 3.23 × 10^−7^), *Thermoleophilia* (*r* = − 0.62, *P* = 1.01 × 10^−2^), and *Gaiella* (*r* = − 0.72, *P* = 1.53 × 10^−3^).

The relative abundance of methanogens (0.43%) as predicted from 16S rRNA analysis had the same correlation with the DOM variance along the first canonical axis (Pearson’s correlation coefficient *r* = 0.93, *P* = 2.16 × 10^−7^), as the genes encoding for methanogenesis (Fig. 5c). Despite their distinctly different substrate range, we found that all methanogenic genera correlated with DOM variance along the first canonical axis (Additional file 8: Table S6). Consistently, both functional ortholog groups (FOAM orthologs) of hydrogenotrophic and aceticlastic methanogenesis showed significant correlation with DOM variance along the first canonical axis (Fig. 5c and Additional file 6: Table S4). Strikingly, methanogenesis via CO_2_ reduction (TMP = 4.54, K00320) had a greater functional potential than aceticlastic methanogenesis (TMP = 1.93, K00193, K00194, K00197) in all paddy sites. Methanogenesis can be performed by syntrophic methanogenic consortia via direct interspecies electron transfer (DIET), mediated by electrically conductive pili (e-pili) or biochar and other conductive materials [25, 26]. So we calculated the Pearson’s correlation coefficient between Fe(III)-reducing bacteria with/without e-pilin encoding genes and the first canonical axis of DOM (Additional file 9: Table S7). Seventeen out of 19 bacteria encoding e-pilin were significantly correlated with DOM variance along the first canonical axis, while 35 out of 70 bacteria without e-pili were also significantly correlated. Consistently, electrically conductive pilin (e-pilin, 46.6%) was significantly positively correlated to DOM compositional change, while long type IVa pilin (53.4%) was not (*r* = 0.61, *P* = 0.013 for e-pilin; *P* > 0.05 for long type IVa pilin).

## Discussion

In this study, we demonstrated that DOM molecular distribution correlates with microbial community structure, taxonomy, and functional potential in paddy soils from sites representing gradients of temperature, precipitation, pH, and human activity. Both molecular distribution of DOM and microbial community structure exhibited significant biogeographic patterns. We considered how biotic and abiotic factors, like geographic distance, MMP, pH, MAT, elevation, and the interactions between microbial communities and DOM molecules, might drive the biogeography of microbial communities and the chemogeography of DOM molecules. These results expand our knowledge of how microbes and DOM molecules are distributed throughout the rice paddy ecosystem.

While variations in the type and abundance of paddy soil DOM molecules have typically been chalked-up to temperature, moisture, pH, and mineralogy [15, 27–29], here, we demonstrated that the microbiome describes DOM variance to a greater extent than any other edaphic or geographic factor investigated. This result emphasizes the overriding potential impact that biotic function factors can have on constructing the DOM heterogeneity when compared to other abiotic functions in paddy soil. The heterogeneous characteristic of DOM is partly attributed to the chemistry of plant-derived compounds and their decomposition byproducts [15, 30, 31]. For instance, genes associated with ligninase or hydrocarbon (mostly aromatics) degradation were positively correlated with polyphenolic and phenolic compounds and polycyclic aromatics in DOM of flooded paddy soils. This indicated biodegradation processes from large biopolymers (e.g., lignin) towards small molecules with concurrent increases in polar and ionizable groups and thus with increase in solubility [5]. Meanwhile, these compounds (known as terrestrial DOM) characterized by high cyclization were selectively coprecipitated with iron at the redox interface during their upward diffusion together with the ferrous ion [3]. Dissimilatory Fe(III)-reducing microbes, e.g., *Geobacter*, were responsible for the production of ferrous ion and thus the coprecipitation of terrestrial DOM with Fe(III) in paddy soil [32, 33]. Many of the DOM compounds may also be derived from microbial residues, e.g., cellular materials and extracellular secretions [34, 35]. Recently, SOM chemistry study in model soils also demonstrated that the accumulation of chemically diverse SOM was driven by distinct microbial communities, per se, rather than the substrates they utilized [36].

Conversely, we also realized that the abundance and distribution of DOM explained variations in the paddy soil microbiome better than any other examined edaphic or geographic factors. The chemical nature of SOM was reported to affect the structure and functioning of paddy soil microbiota [6, 37]. In our study, more biodegradable substances, e.g., peptides and carbohydrates, favored microbes (e.g., *Geobacter*) utilizing mainly simple C forms and functions regarding glycolysis (EMP) and TCA cycle in the flooded paddy. This heightened concentration of biodegradable substances also facilitated pyruvate fermentation, and associated metabolic processes, e.g. methanogenesis and homoacetogenesis [38]. Microbial consortia that cooperatively exchange electrons were pivotal in the anaerobic processing of SOM [25, 26]. Therefore, the increase of Fe(III)-reducing bacteria encoding e-pilin may promote the propagation of hydrogenotrophic methanogens. Consistently, we found that gene encoding e-pilin, as a potential indicator of the DIET [39, 40], was significantly correlated with DOM variance. Moreover, a recent study proved that the aceticlastic methanogen *Methanothrix* spp. receives electrons for the reduction of CO_2_ via DIET from microorganisms expressing e-pilin genes, e.g., *Geobacter* [39]. Decreased abundance of DOM compounds with quinone moieties, which were likely derived from polyphenolic and phenolic compounds and polycyclic aromatics, reduced the possibility that electrons are being transferred to these DOM and used in Fe(III) reduction [41, 42], thus increasing the electrons available for methanogenesis.

Of the abiotic factors, precipitation (MMP), temperature (MAT), and pH explained the majority of the main variance in DOM composition across the continental scale paddy fields. pH and temperature have also been reported to drive soil microbial community composition at continental scales [10, 11]. Here, we have found that temperature (MAT) and pH were significantly correlated with the covariations between DOM and microbial community. Therefore, the influences of pH and temperature on DOM diversity may be mediated by microbial community. Consistently, pH was not strongly correlated with the majority of individual DOM molecules (Additional file 1: Figure S3), but it could explain the DOM component variance, implying an indirect effect on DOM molecular distribution. Elevated temperature may stimulate the biodegradation of plant residues, but the consequences for microbial-derived residues are less clear [11, 36].

Precipitation shapes DOM chemodiversity as it enhances upward movement of ferrous ions and DOM molecules from deep soils, and influences the influx of selective DOM from the surface soil. Precipitation events dilute not only the ion and DOM concentrations of the standing water per se, but also that of the irrigation water sources (i.e., the nearby bodies of water), enhancing the upward movement of Fe(II) and DOM from deep soils [43]. Meanwhile, terrestrial DOM would be selectively trapped at the Ap horizon (oxic and partly oxic) via coagulation with the precipitating Fe(III) during the upward diffusion [1, 3, 44], which then accelerates the upward diffusion of these compounds. Moreover, terrestrial DOM is relatively harder to be regenerated when compared to carbohydrate and peptide [45]. Besides, abundant rainfall preserves the gradient in reduction potential across depth, which increases not only the mineral reduction but also the opportunity of DOM reduction in paddy fields [1, 6, 43]. As evidence, significant correlations were found between precipitation (MMP) and genes encoding caa3-type and bo-type cytochrome oxidases (Fig. 5b) and cellular response to oxidative stress (Additional file 7: Table S5). The caa3-type and bo-type cytochrome oxidases are mostly found in aerobes, while cbb3-type and bd-type cytochrome oxidases have been reported to be utilized by anaerobes or microaerophiles in microaerobic energy metabolism [46, 47]. In this case, precipitation may also shape microbial community.

Factors like parential metrial, redox state, fertilization level, pesticide application, mineralogy, rice cultivar, and growth stage may also influence the geography of the microbial community and DOM [29, 48–53]. In submerged paddy soil, rice aerenchyma enables the transport of atmospheric O_2_ to the roots [54], influencing soil redox states [55]; moreover, rice straw and stubble are assumed to provide substrates for microbial activity in the early growth stage, while exudates become more important during late tillering and ripening [56, 57]. Therefore, the effect of the rice plant on the soil microbial community largely depends on the plants growth stage. In this study, the tiller number of the rice (indicating the growth stage) was significantly correlated with DOM variance (PCo1) and DOM features (Fig. 3e). Recently, a relatively comprehensive study on the DOM chemodiversity of paddy soil and factors, including mineral elements of Fe, Mn, Al, Mg, Ca, and Si, demonstrated that the iron complexing index (Fe_p_/Fe_R_), together with pH and C/N ratio, were key factors controlling DOM profiles [29]. Another study on agricultural, meadow, and forest soils revealed that pH and nitrate significantly affect the chemical composition of DOM molecules [14]. These factors were also separately found to significantly correlate with the microbiome in paddy soils [48, 58, 59]. Although rice cultivation management dominated the microbial community assembly in paddy soils [60], the soil parent material was also influential [58], and hence, the influence on DOM distribution should be further investigated. Although these previous studies and the current one presented here principally focus on the spatial distribution of the microbiome and DOM molecules, how these patterns change over time is also important [61]. Redox potential is one of the key temporal factors and is controlled by soil ventilation [1]. Temporal changes of irrigation management, precipitation, and even light intensity may quickly change the redox condition in soils. Researchers have revealed that redox potential could significantly shift microbial community composition [6, 53]. Retention of certain DOM molecules by soil minerals and their subsequent stabilization against microbial decay were also largely dependent on the redox state [3, 5]. However, it remains unknown whether the temporal dynamics of microbial communities correlates shifts in DOM composition. Therefore, there is a continued need for new, well-controlled studies to further elucidate DOM chemogeography and microbial biogeography.

## Conclusions

Understanding the relationship between soil DOM and microbial community structure and function remains a research goal for biogeochemists, especially at the molecular level [8, 52]. Here, we integrated mass spectra and genomics data to characterize the association between DOM molecular distribution and microbial diversity and applied gene-centric analysis to elucidate the microbial metabolic potential that responds and shapes DOM heterogeneity. DOM chemodiversity was significantly and broadly correlated with the taxonomy and functional potential of the microbial community in paddy soil. Besides pH and temperature, precipitation was also found to be a potential factor of microbial community and DOM chemical distribution. These findings are foundational, but could be of great importance for environmental and agricultural management in paddy soils.

## Methods

### Site selection and soil sampling

Soil samples were collected from 88 flooded paddy sites across four typical Chinese rice-growing regions in 2014 and 2015 (Fig. 1). Most of the soils were sampled during the tillering phase of rice plants in the paddy fields. Among 88 sampling sites, there were 23 from Hani Terrace, 24 from Sanjiang Plain, 18 from Lianghu Plain, and 23 from Taihu Plain (Table 1). At each site, soil cores (2.5 cm diameter by 15 cm depth) were sieved (2 mm) and homogenized, and plant materials were removed immediately before sealing and transportation. For more details about soil sampling and characteristics measurements (Additional file 10: Table S8), please see supplementary methods in Additional file 11.

### FT-ICR-MS data analysis

FT-ICR-MS samples were prepared and measured according to Kellerman et al. [4] with some modifications (for details see supplementary methods in Additional file 11). Detected peaks were considered if the signal-to-noise ratio was greater than five. After calibration, different spectral peaks were clustered into operational units within a mass tolerance with m/z difference ratios less than 1 × 10^−6^. The detailed methods for calibration and clustering are described in Additional file 11. Clusters with fewer than ten peaks were not considered for further annotation.

Based on the two mandatory and two optional steps for peak annotation by Koch et al. [16], we introduced a carbon isotope ratio-based molecular annotation approach (Additional file 1: Figure S5), in which molecular formulae are assigned to peaks according to stringent criteria with elemental combinations of C_1-100_H_1-150_O_0-50_N_0-4_P_0-1_S_0-1_. The isotope-based approach first tries to find the carbon isotope peak of a certain large peak according to mass differences and then calculates the potential C number in the molecular formula based on the relatively stable ratio of naturally occurring ^13^C-isotope to ^12^C (i.e., 1.07%) and the intensity ratio of the two peaks. At the same time, several alternative formulas are calculated according to an a priori definition of elements and unequivocal exclusion criteria. Then, the carbon numbers of these formulas are subtracted by the potential C number for carbon number differences (C_dev_), and the molecular formula with the smallest C_dev_ is chosen. In this study, C_dev_ was defined as acceptable when the C_dev_ was (− 3, 1) [16]. Annotated formulas were then used as a scaffold, and a “chemical building block” approach was adopted to annotate the rest of the peaks. Meanwhile, the relevant ^13^C, ^15^N, ^34^S, ^33^S, ^18^O, ^17^O, ^2^H, ^13^C_2_, ^13^C_3_, and ^13^C^34^S isotope molecules were all determined, if detected.

The annotated molecules were assigned to compound categories based on the stoichiometry of their molecular formulas [45]: polycyclic aromatics (aromaticity index, AI > 0.66) [62], polyphenols and polycyclic aromatics with aliphatic chains (0.66 ≥ AI > 0.50), phenolic and highly unsaturated/aromatic compounds (AI ≤ 0.50 and H/C ≤ 1.5), unsaturated aliphatics and aromatics with aliphatic chains (*N* = 0, AI ≤ 0.50, 2.0 ≥ H/C ≥ 1.5), saturated fatty, sulfonic acids and carbohydrates (H/C ≥ 2.0 or O/C ≥ 0.9), and N-containing compounds, i.e., peptides (*N* ≥ 1, AI ≤0.50, 2.0 ≥ H/C ≥ 1.5).

### 16S rRNA sequencing and analysis

DNA was extracted using the MoBio PowerSoil DNA extraction kit following the manufacturer’s protocol. The concentration and qualification of the total DNA were examined by electrophoresis on 1% agarose gels, which was diluted 1 ng/μL using sterile water before downstream processing. PCR procedure was carried out as described previously [63]. Briefly, the V4-V5 region of the 16S rRNA gene from each soil sample was amplified using the F515/R907 primer set with a unique 6-nt barcode at the 5′ of the forward primer [64]. All PCR reactions were carried out with Phusion® High-Fidelity PCR Master Mix (New England Biolabs). After electrophoresis on 2% agarose gel, PCR products with bright main strip between 400 and 450 bp were mixed in equidensity ratios and purified with Qiagen Gel Extraction Kit (Qiagen, Germany). Sequencing libraries were generated using the TruSeq® DNA PCR-Free Sample Preparation Kit (Illumina, USA) following the manufacturer’s recommendations, and index codes were added. After quality assessing, the libraries were sequenced on an Illumina HiSeq2500 (Novogene, China), and 250 bp paired-end reads were generated.

The raw sequence data were processed using the QIIME v1.9.1 pipeline [65]. Firstly, the forward and reverse Illumina reads were joined using the default setting. Then, the multi-lane fastq data were demultiplexed and quality filtered (Q30 ≥ 75% and Q20 = 100%). Chimeras were identified using “identify_chimeric_seqs.py” with “-m usearch61” and then removed. A total of 15,339,665 reads were kept with a number over 43,000 for each sample. Filtered sequences were clustered into operational taxonomic units (OTUs) using the function “pick_open_reference_otu.py” against the SILVA 119 database [66], based on a 97% consensus threshold. Then, the singletons were removed and taxonomy was assigned using the RDP classifier against the SILVA database. R package Tax4fun [24] with UProC long read mode was used to predict the functional capabilities of microbial community in each sample based on assigning OTUs of 16S rRNA gene to the reference sequences in the SILVA (version 119, 97 set) database via SortMeRNA [67]. HUMAnN2 [68] was used to map the resulted orthologs to functional ontology of FOAM database [69].

### Shotgun sequencing, metagenome assembly, and annotation

A sum of 16 samples (4 samples from each region) was randomly selected for shotgun metagenome sequencing. Total DNA was extracted using the same method as that of 16S rRNA sequencing. DNA concentration was measured using Qubit® dsDNA Assay Kit in Qubit® 2.0 Flurometer (Life Technologies, USA). Using 1 μg DNA per sample as input material, sequencing libraries were generated using NEB Next® Ultra™ DNA Library Prep Kit (NEB, USA), and index codes were added to attribute sequences to each sample. Briefly, the DNA was broken into 350 bp fragments using sonication, polished and extracted using the AMPure XP system. Libraries were prepared on a cBot Cluster Generation System following the manufacturer’s instructions. After cluster generation, the library preparations were sequenced on an Illumina HiSeq platform (Novogene, China) and 150 bp paired-end reads were generated.

Paired-end reads were quality controlled using Readfq v8 (https://github.com/cjfields/readfq): sequences with more than 40 bases, with quality score lower than 38, or with N bases more than 10 were filtered. The adapter was also removed from the sequences. Metagenome sequencing yielded about 12.8 G clean bases per sample (Additional file 12: Table S9). Taxonomical classification of the sequencing reads of each sample was performed using Kaiju [70] with greedy-5 mode against an nr-derived database including proteins from archaea, bacteria, viruses, fungi, and microbial eukaryotes. Based on the classification result, relative abundances of Fe(III)-reducing bacteria with or without e-pili were calculated (Additional file 9: Table S7). The 93 known Fe(III)-reducing microorganisms for which genomes are available were obtained from a previous study [39]. Metagenome assemblies were conducted for each sample using MEGAHIT v1.1.1 [71] with “--presets meta-large.” Metagenome assemblies yielded a total of 41.8 M contigs over 200 bases length and 10.1 M contigs over 500 bases length (average length was 458 bases; Additional file 13: Table S10). A total of 53.5 M nucleotide sequences or protein translations of genes were predicted from the contigs (≥ 200 bp) of each sample using prodigal [72] with “-p meta.” These genes or proteins were then pooled together to form a gene catalog and a protein catalog, respectively. The protein catalog was annotated using the FOAM database [69] and hmmer3.1 [73] to obtain function orthologs as defined in KEGG Orthology [74]. We chose profile’s trusted cutoffs to set all thresholding. The resulted orthologs were then mapped to an associated functional ontology of FOAM database [69] to describe the functional groups and hierarchy. Metagenomic contigs were annotated to gene and enzyme using prokka pipeline [75]. The marker genes used in the analyses of biogeochemical functions were selected from a hidden markov model database [76] with a few modifications. Pfam [77] and TIGRfam [78] protein families were assigned using hmmer3.1 [79]. Profile’s trusted cutoffs were used as thresholds. The presence of type IV pilA genes was estimated by assigning the gene catalog to nucleotide sequence database with 33 e-pilin genes and 27 long pilin genes [40] using Diamond [80] with parameters set as following: --more-sensitive, -e 0.00001, -l 20. For each gene, the best blast hit (one HSP > 60 bits) result was selected for downstream analyses. To quantify the annotated genes in each sample, we mapped the paired-end reads back to the assemblies according to the pipeline described here: http://metagenomics-workshop.readthedocs.org/, together with the other tools, i.e., bowtie2 [81], samtools [82], and htseq [83]. As suggested by the pipeline, we used the TPM (Transcripts Per Kilobase Million) method [84] to normalize abundance values in metagenomes. The gene encoding for acetyl-CoA synthetase (K01895) is normally multi-copy, so was not chosen for the comparison.

### DOM and bacterial diversity calculations and multivariate analysis

Accumulation and rank abundance curves of DOM were calculated with the sum-normalized intensity of non-singleton data using R package *Biodiversity R* [85] and *vegan* [86]. Bray-Curtis dissimilarity was used to compute the sparse matrices of DOM molecules and bacterial community. The molecular and bacterial alpha diversities and beta diversities were calculated using QIIME v1.9.1 [65]. VAW-UniFrac dissimilarity was calculated using R package *GUniFrac* [87]. Modified R function kde2d weighted from kde2d in *MASS* package [88] was used to perform two-dimensional kernel density estimation with an axis-aligned bivariate normal kernel in van Krevelen diagram for the density of DOM molecules in each sampling region. Median values of molecular abundances were defined as the weights parameter. Mantel test was conducted to determine whether two distance matrixes were significantly correlated. PERMANOVA test was conducted to determine whether DOM molecular Bray-Curtis dissimilarity was significantly different between regions. CCA and NMDS were performed using rounded intensities rarefied at the depth of 43,000 for both DOM and bacterial communities (or their dissimilarity matrices). Partial CCA was used to calculate the independent influences of different categories or parameters on DOM or microbial variance. Principal coordinates analysis (PCoA) was used to calculate the gradient in compositional changes of microbial community (based on Bray-Curtis or VAW-UniFrac), DOM (based on Bray-Curtis), and different DOM categories (based on Bray-Curtis). Procrustes rotation and Monte Carlo permutation test (permutation = 9999) were performed using the two coordinate matrices (output of PCoA) based on their Bray-Curtis dissimilarities. We performed the test using the first ten axes of DOM and bacterial coordinate matrices (explained 67% and 65% variations, respectively). CCorA was conducted using PCo1–5 axes of DOM composition and PCo1–5 axes of microbial community composition or PCo1–2 axes of functional orthologs distance matrices (Bray-Curtis dissimilarities) as imports following the procedure described by Osterholz et al. [19]. These analyses were all conducted using *vegan* package [86].

## Additional files

Additional file 1: Alpha-diversities of bacterial OTUs and DOM molecules in the tested soil samples across four typical paddy fields. **A**: Boxplots of alpha-diversities of bacterial OTUs using four indices: Chao1, observed species, Shannon and PD whole tree. **B**: Boxplots of alpha-diversities of DOM molecules using three indices: Chao1, observed species, and Shannon. We randomly subsampled 43,000 sequences and 9000 per sample ten times to correct for differences in sequencing depth for bacterial OTUs and DOM molecules. In the boxplots, the symbols indicate the following: box, lower and upper quartiles; horizontal line, median value; whiskers, lower and upper inner fence. The circle above or below the box plots indicates outliers. Differences among the four regions were tested using non-parametric Kruskal–Wallis test (*P* < 0.05). Paired boxes containing no same letter are considered to be significantly different (Dunn’s test, *P* < 0.05). Figure S2 Boxplots of regional differences of top taxa classified using 16S rRNA gene. Differences among the four areas were tested using non-parametric Kruskal–Wallis test followed by Dunn’s test for pairwise multiple comparisons. **A**: Regional differences of the top 10 phyla. **B**: Regional differences of the top 10 classes. **C**: Regional differences of the top 15 orders. **D**: Regional differences of the top 20 families. In the boxplots, the symbols indicate the following: boxes, the interquartile range (IQR) between first and third quartiles; horizontal line, median value; whiskers, the ranges of lower and higher values within 1.5 × IRQ from the first and the third quartiles, respectively; circles, outliers beyond the whiskers; *, *P* < 0.05; **, *P* < 0.01; ***, *P* < 0.001. Figure S3 Van Krevelen plots of DOM molecules, showing Spearman’s rank correlations with the factors used in Fig. 3e. Only DOM molecules with “BH” FDR-adjusted *P* ≤ 0.05 and |*ρ*| ≥ 0.3 are shown here. Strong correlations (|*ρ*| ≥ 0.5) were indicated by black perimeter. Category A: saturated fatty and sulfonic acids, carbohydrates; category B: N-containing compounds, i.e., peptides; category C: unsaturated aliphatic compounds, aromatic hydrocarbon; category D: phenolic and highly unsaturated compounds; category E: polyphenols and polycyclic aromatics (PCAs) with aliphatic chains; category F: combustion-derived PCAs. Figure S4 Comparisons analysis of the relative abundances of dominant genera estimated by 16S rRNA and metagenomics. Fifteen dominant genera are shown here with each plot showing the comparison for a specific genus. The classified taxa of fungi, viruses, and microbial eukaryotes of metagenomic data are not considered here. Samples from Sanjiang Plain: H02, H18, H22, H47; samples from Lianghu Plain: L01, L07, L20, L28; samples from Taihu Plain: T09, T17, T31, T48; samples from Hani Terrace: Y04, Y24, Y30, Y43. Pearson’s correlation coefficients (*ρ*) and statistical significances (*P*) are inscribed in each plot. Figure S5 Flowchart of in-house software for the annotation of DOM molecules. Unequivocal exclusion criteria (elements should follow these rules): C > 0; *N* ≥ 0; H > 0; O ≥ 0; 1 ≥ *P* ≥ 0; 1 ≥ S ≥ 0; H ≥ C/3; H ≤ 2C + N + P + 2; 2|(N + H + P) = 0; N ≤ C; O + S ≤ C + 2 N + 3P; O + S ≥ P. Functional group relationships or “chemical building block” used in elemental formula assignment: CH_4_ - O (0.036385 Da), C_2_H_2_ (26.015650 Da), C_2_H_4_ (28.031300 Da), CH_2_ (14.015650 Da), H_2_ (2.015650 Da), H_2_O (18.010565 Da), O (15.994915 Da), CO_2_ (43.989830 Da), NH (15.010899 Da), S (31.972071 Da). (DOCX 1840 kb)

Additional file 2: **Table S1.** Statistics of DOM extraction and annotation of soil samples collected in this study. Peak number a = number of peaks after filtering blank peaks and singletons. Peak number b = number of peaks after filtering blank peaks and those present in less than 10 samples, which is further introduced to annotation procedure. Peak number c = number of peaks assigned to putative formulas. (XLSX 18 kb)

Additional file 3: **Table S2.** Multivariate analysis of bacterial data and drivers using canonical correspondence analysis (CCA) and non-metric multidimensional scaling (NMDS). Constrained (CCA) and unconstrained methods (NMDS and envfit) were used to compare and interpret effects of edaphic, geographical, and DOM factors on the microbial diversity. CCA1, CCA2, MDS1, and MDS2 stand for the angle cosines of variables and the axes. Pvals.1, empirical *P* values of fit statistic of environmental variables in CCA; Pvals.2, empirical *P* values of fit statistic using envfit to present environmental variables in NMDS; *r*^2^, goodness of fit statistic. Variables with Pvals.2 ≤ 0.001 were ticked on the “Fig. 3c” column and shown on Fig. 3c. (XLSX 16 kb)

Additional file 4: **Table S3.** Multivariate analysis of DOM data and drivers using canonical correspondence analysis (CCA) and non-metric multidimensional scaling (NMDS). Constrained (CCA) and unconstrained methods (NMDS and envfit) were used to compare and interpret effects of edaphic, geographical, and bacterial factors on the DOM chemodiversity. CCA1, CCA2, MDS1, and MDS2 stand for the angle cosines of variables and the axes. Pvals.1, empirical *P* values of fit statistic of environmental variables in CCA; Pvals.2, empirical *P* values of fit statistic using envfit to present environmental variables in NMDS; *r*^2^, goodness of fit statistic. Variables with Pvals.2 ≤ 0.001 were ticked on the “Fig. 3d” column and shown on Fig. 3d. Variables used in Fig. 3e were ticked on the “Fig. 3e” column. (XLSX 16 kb)

Additional file 5: Supplementary results about consistence between metagenomic data and 16S rRNA data. (DOCX 29 kb)

Additional file 6: **Table S4.** Significant correlations between predicted functions and the compositional changes of DOM. Pearson’s correlation coefficient tests were performed between predicted FOAM functions (level 1 and 2) and the compositional changes of DOM along first and second canonical axes obtained from canonical correlation analysis (CCorA). CCorA was performed using the first five principal coordinate (PCo1–5) axes of DOM composition and PCo1–2 axes of FOAM orthologs. It resulted in two canonical axes (*P* < 0.01, chi-square test) along which significant correlations were observed between functional potentials and DOM composition (*P* < 0.013). Only functions with significant correlations (*P* < 0.05) were shown here. (XLSX 21 kb)

Additional file 7: **Table S5.** FOAM orthologous groups used for comparing metagenomes with Tax4fun-predicted functions. Pearson’s correlation coefficient was used for the correlation estimation. Only orthologous groups significantly correlated with DOM variations were tested here. (XLSX 11 kb)

Additional file 8: **Table S6.** Significant correlations between major methanogen genera in the metagenomic samples and the compositional change of DOM. Pearson’s correlation coefficient tests were performed between major taxonomic groups of methanogens in the metagenomic samples and the compositional change of DOM along first canonical axis obtained from canonical correlation analysis (CCorA). CCorA was performed using the first five principal coordinate (PCo1–5) axes of DOM composition and PCo1–2 axes of FOAM orthologs. It resulted in two canonical axes (*P* < 0.01, chi-square test) along which significant correlations were observed between functional potentials and DOM composition (*P* < 0.013). Only taxa with significant correlations *(P* < 0.05) were shown here. MeNH_2_ is methylamine. Parentheses means utilized by some, but not all species or strains. (XLSX 12 kb)

Additional file 9: **Table S7.** Pearson’s correlation coefficients between Fe(III)-reducing microorganisms in the metagenomic samples and the compositional change of DOM. Information of the type IVa pilA genes was given for their potential extracellular electron transport mechanisms. Pearson’s correlation coefficient and *P* value were correlated using the abundance of each species and the first canonical axis of DOM variation. (XLSX 16 kb)

Additional file 10: **Table S8.** Soil and site characteristics associated with each of the soil samples collected in this study. MAT = mean annual temperature. MAP = mean annual precipitation, MMT = mean monthly temperature, MMP = mean monthly precipitation (the data we used is the month data we sampled at that site or the mean value of 2-month data if the sampling date was at the beginning or the end of a month), RST = real-time soil temperature, RAT = real-time air temperature, DOC = dissolved organic carbon, Olsen-P = rapid available phosphorus, Ph = rice plant height, FD = flooding depth. MAT, MAP, MMT, and MMP were 22-years observation date obtained from http://neo.sci.gsfc.nasa.gov. The total carbon (TC), total nitrogen (TN), and total phosphorus (TP) were on dry basis while the RAP was on wet basis. Field moisture was measured immediately after sample collection. (XLSX 25 kb)

Additional file 11: Supplementary methods. (DOCX 32 kb)

Additional file 12: **Table S9.** Basic information of the metagenomic sequencing. (XLSX 11 kb)

Additional file 13: **Table S10.** Statistics information of metagenomic assembly for each metagenomic sample. (XLSX 10 kb)

## Abbreviations

AI: Aromaticity index
CCA: Canonical correspondence analysis
CCorA: Canonical correlation analysis
DIET: Direct interspecies electron transfer
DOM: Dissolved organic matter
EMP: Embden-Meyerhof-Parnos
e-pili: Electrically conductive pili
e-pilin: Electrically conductive pilin
FOAM: Functional Ontology Assignments for Metagenomes
FT-ICR-MS: Fourier transform ion cyclotron resonance mass spectrometry
MAT: Mean annual temperature
MMP: Mean monthly precipitation
NMDS: Non-metric multidimensional scaling
OTU: Operational taxonomic unit
PCo: Principal coordinate
PCoA: Principal coordinate analysis
PERMANOVA: Permutational multivariate analysis of variance
SOM: Soil organic matter
TCA: Tricarboxylic acid
TPM: Transcripts Per Kilobase Million
VAW-UniFrac: Variance adjusted weighted UniFrac
