# Correction to: The chemodiversity of paddy soil dissolved organic matter correlates with microbial community at continental scales

**DOI:** 10.1186/s40168-020-00945-3

**Published:** 2020-11-28

**Authors:** Hong-Yi Li, Hang Wang, Hai-Tao Wang, Pei-Yong Xin, Xin-Hua Xu, Yun Ma, Wei-Ping Liu, Chang-Yun Teng, Cheng-Liang Jiang, Li-Ping Lou, Wyatt Arnold, Lauren Cralle, Yong-Guan Zhu, Jin-Fang Chu, Jack A. Gilbert, Zhi-Jian Zhang

**Affiliations:** 1 College of Environment and Natural Resource Sciences, Zhejiang University, 866 Yuhangtang Ave, Hangzhou, 310058 China; 2 National Plateau Wetlands Research Center, Southwest Forestry University, 300 Bailongsi, Kunming, 650224 China; 3 The Microbiome Center, Biosciences Division, Argonne National Laboratory, Lemont, IL 60439 USA; 4 Department of Surgery, University of Chicago, 5640 South Ellis Avenue, Chicago, IL 60637 USA; 5 National Center of Plant Gene Research (Beijing), Institute of Genetics and Developmental Biology, Chinese Academy of Sciences, West Beichen Road, Chaoyang District, Beijing, 100101 China; 6 College of Biological and Environmental Engineering, Zhejiang University of Technology, 18 Chaowang Ave, Hangzhou, 310014 China; 7 Hangzhou Gusheng Agricultural Technology Company Limited, Chongxian Innovation Industrial Park, Chongxian Ave, Hangzhou, 311108 China; 8 Key Lab of Urban Environment and Health, Institute of Urban Environment, Chinese Academy of Sciences, 1799 Jimei Ave, Xiamen, 361021 China; 9 China Academy of West Region Development, Zhejiang University, 866 Yuhangtang Ave, Hangzhou, 310058 China

**Correction to: Microbiome 6, 187 (2018)**

**https://doi.org/10.1186/s40168-018-0561-x**

Following publication of the original article [1], the authors identified an error in the name of the third author.

The incorrect author name is: Hai-Tiao Wang

The correct author name is: Hai-Tao Wang

The author group has been updated above and the original article [1] has been corrected.

## References

[1] Li HY, Wang H, Wang HT (2018). The chemodiversity of paddy soil dissolved organic matter correlates with microbial community at continental scales. Microbiome.

