# Supplementary material for: The chemodiversity of paddy soil dissolved organic matter correlates with microbial community at continental scales
Source: Microbiome. 2018 Oct 19;6:187. doi: 10.1186/s40168-018-0561-x (PMC6195703; doi:10.1186/s40168-018-0561-x)
Supplement: Supplementary file 1 — Alpha-diversities of bacterial OTUs and DOM molecules in the tested soil samples across four typical paddy fields. A: Boxplots of alpha-diversities of bacterial OTUs using four indices: Chao1, observed species, Shannon and PD whole tree. B: Boxplots of alpha-diversities of DOM molecules using three indices: Chao1, observed species, and Shannon. We randomly subsampled 43,000 sequences and 9000 per sample ten times to correct for differences in sequencing depth for bacterial OTUs and DOM molecules. In the boxplots, the symbols indicate the following: box, lower and upper quartiles; horizontal line, median value; whiskers, lower and upper inner fence. The circle above or below the box plots indicates outliers. Differences among the four regions were tested using non-parametric Kruskal–Wallis test (P < 0.05). Paired boxes containing no same letter are considered to be significantly different (Dunn’s test, P < 0.05). Figure S2 Boxplots of regional differences of top taxa classified using 16S rRNA gene. Differences among the four areas were tested using non-parametric Kruskal–Wallis test followed by Dunn’s test for pairwise multiple comparisons. A: Regional differences of the top 10 phyla. B: Regional differences of the top 10 classes. C: Regional differences of the top 15 orders. D: Regional differences of the top 20 families. In the boxplots, the symbols indicate the following: boxes, the interquartile range (IQR) between first and third quartiles; horizontal line, median value; whiskers, the ranges of lower and higher values within 1.5 × IRQ from the first and the third quartiles, respectively; circles, outliers beyond the whiskers; *, P < 0.05; **, P < 0.01; ***, P < 0.001. Figure S3 Van Krevelen plots of DOM molecules, showing Spearman’s rank correlations with the factors used in Fig. 3e. Only DOM molecules with “BH” FDR-adjusted P ≤ 0.05 and |ρ| ≥ 0.3 are shown here. Strong correlations (|ρ| ≥ 0.5) were indicated by black perimeter. Category A: saturated fatty and s [file 40168_2018_561_MOESM1_ESM.docx]

**Supplementary Figures:**

**Figure S1**


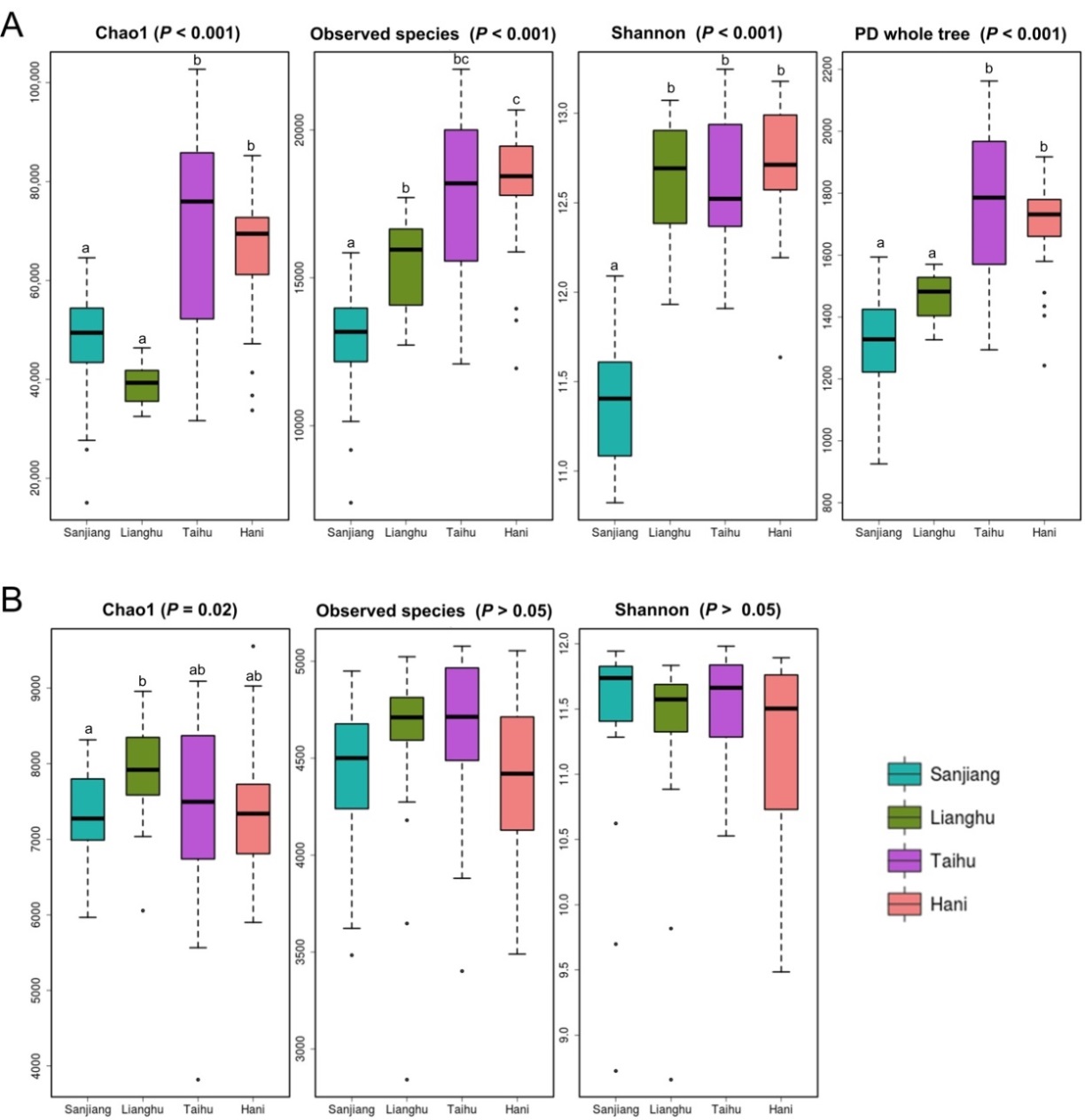


**Supplementary Figure 1.** **Alpha-diversities of soil samples across four typical paddy fields. A:** Boxplots of alpha-diversities of bacterial OTUs using four indices: Chao1, Observed species, Shannon and PD whole tree. **B:** Boxplots of alpha-diversities of DOM molecules using three indices: chao1, Observed species and Shannon. We randomly subsampled 43,000 sequences and 9,000 per sample ten times to correct for differences in sequencing depth for bacterial OTUs and DOM molecules. In the boxplots, the symbols indicate the following: box, lower and upper quartiles; horizontal line, median value; whiskers, lower and upper inner fence. The circle above or below the box plots indicates outliers. Differences among the four areas were tested using non-parametric Kruskal–Wallis test (*P* < 0.05). Paired boxes containing no same letter are considered to be significantly different (Dunn test, *P* < 0.05).

**Figure S2**


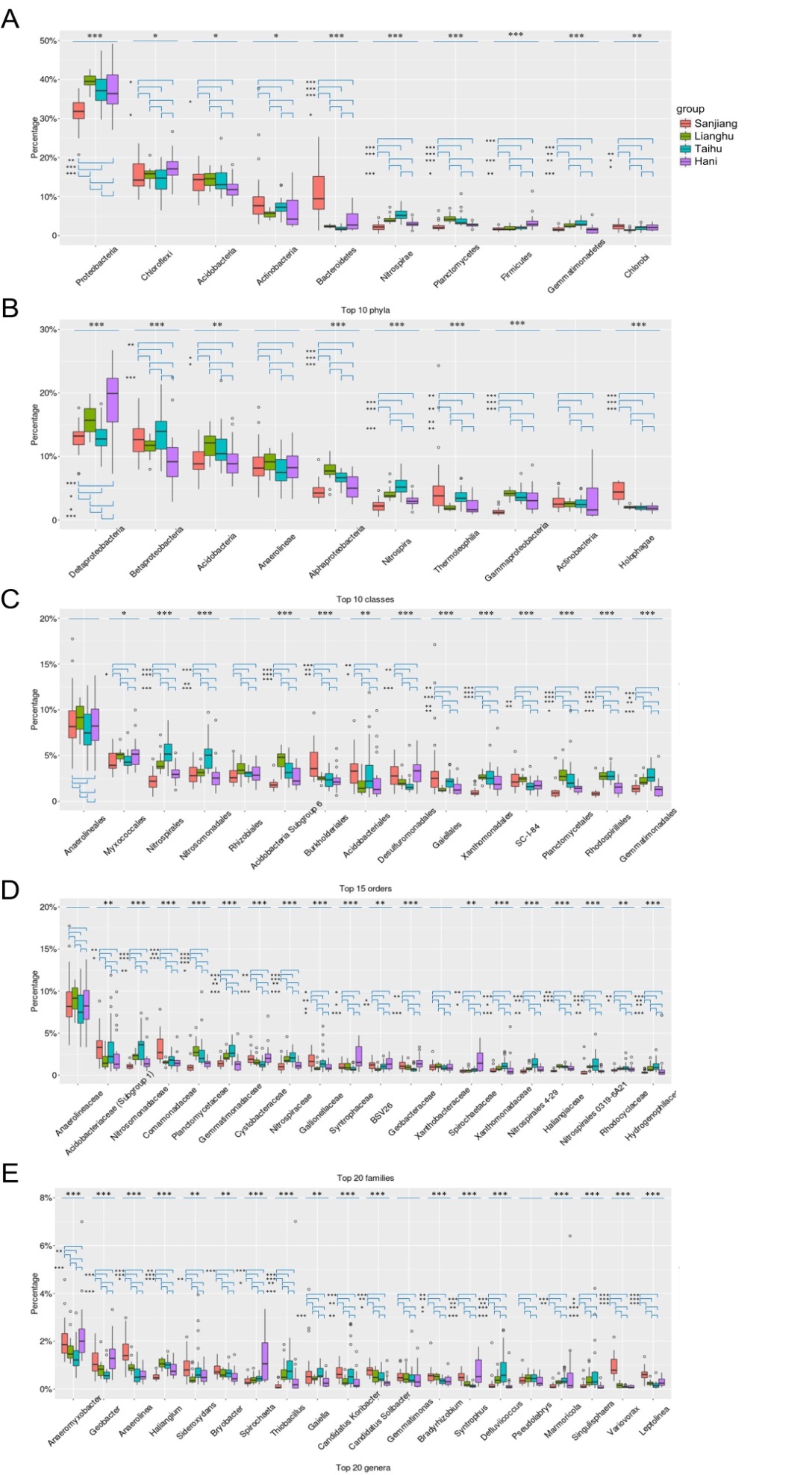


**Supplementary Figure 2. Boxplots of regional differences of top taxa classified using 16S rRNA gene.** Differences between the four regions were tested using Kruskal–Wallis test followed by Dunn’s test. **A**: Regional differences of the top 10 phyla. **B**: Regional differences of the top 10 classes. **C**: Regional differences of the top 15 orders. **D**: Regional differences of the top 20 families. In the boxplots, the symbols indicate the following: boxes, the interquartile range (IQR) between first and third quartiles; horizontal line, median value; whiskers, the ranges of lower and higher values within 1.5 × IRQ from the first and the third quartiles, respectively; circles, outliers beyond the whiskers; *, P < 0.05; **, P < 0.01; ***, P < 0.001.

**Figure S3**





**Supplementary Figure 3. Van Krevelen plots of DOM molecules, showing Spearman’s rank correlations with the factors used in Fig. 3e**. Only DOM molecules with “BH” FDR-adjusted *P* ≤ 0.05 and |*ρ*| ≥ 0.3 are shown here. Strong correlations (|*ρ*| ≥ 0.5) were indicated by black perimeter. Category A: saturated fatty and sulfonic acids, carbohydrates; Category B: N-containing compounds, i.e. peptides; Category C: unsaturated aliphatic compounds, aromatic hydrocarbon; Category D: phenolic and highly unsaturated compounds; Category E: polyphenols and polycyclic aromatics (PCAs); Category F: combustion-derived PCAs.

**Figure S4**





**Supplementary Figure 4. Comparisons analysis of the relative abundances of dominant genera estimated by 16S rRNA and metagenomics.** 15 dominant genera are shown here with each plot showing the comparison for a specific genus. The classified taxa of fungi, viruses and microbial eukaryotes of metagenomic data are not considered here. Samples from Sanjiang Plain: H02, H18, H22, H47; Samples from Lianghu Plain: L01, L07, L20, L28; Samples from Taihu Plain T09, T17, T31, T48; Samples from Hani Terrace: Y04, Y24, Y30, Y43. Pearson’s correlation coefficients (*ρ*) and statistical significances (*P*) are inscribed in each plot.

**Figure S5**





**Supplementary Figure 5. Flowchart of in-house software for the annotation of DOM molecules.** Unequivocal exclusion criteria (elements should follow these rules): C > 0; N ≥ 0; H > 0; O ≥ 0; 1 ≥ P ≥ 0; 1 ≥ S ≥ 0; H ≥ C/3; H ≤ 2C + N + P +2; 2|(N + H + P) = 0; N ≤ C; O + S ≤ C + 2N + 3P; O + S ≥ P. Functional group relationships or “chemical building block” used in elemental formula assignment: CH_4_ - O (0.036385 Da), C_2_H_2_ (26.015650 Da), C_2_H_4_ (28.031300 Da), CH_2_ (14.015650 Da), H_2_ (2.015650 Da), H_2_O (18.010565 Da), O (15.994915 Da), CO_2_ (43.989830 Da), NH (15.010899 Da), S (31.972071 Da).
