# Supplementary material for: The chemodiversity of paddy soil dissolved organic matter correlates with microbial community at continental scales
Source: Microbiome. 2018 Oct 19;6:187. doi: 10.1186/s40168-018-0561-x (PMC6195703; doi:10.1186/s40168-018-0561-x)
Supplement: Supplementary file 5 — Supplementary results about consistence between metagenomic data and 16S rRNA data. (DOCX 29 kb) [file 40168_2018_561_MOESM5_ESM.docx]

**Supplementary Results**

***Consistence between metagenomic taxa and 16s rRNA taxa***

15 dominant genera were tested for consistence between relative abundances of genera estimated by 16S rRNA data analysis and metagenomic fragments in 16 samples (see Figure A below in this file). The classified taxa of fungi, viruses and microbial eukaryotes of metagenomic data were not considered here. The relative abundances of many genera were significantly different between the 16S rRNA and metagenomic datasets (e.g *Candidatus Solibacter* and *Bradyrhizobium*). This was because the relative abundance of taxa inferred from 16S rRNA was in denomination of 16S rRNA copy numbers, while metagenomic taxonomic classification was in denomination of the abundance of assigned reads. Pearson’s correlation coefficient analysis between amplicon and metagenomic data revealed both high correlation (11 of 15; e.g. *Geobacter*, *Haliangium*, *Sideroxydans* and *Bryobacter*), and no correlation (4 of 15; e.g. *Anaeromyxobacter* and *Thiobacillus*). These may reflect not only the bias in the 16S sequencing approach [1], but also the sampling bias in the phylogenetic distribution of available reference genomes for metagenomic taxonomic classification [2].

***Consistence between metagenomic functional analysis and 16s rRNA based functional prediction***

To confirm the findings from the limited metagenomic analysis, Tax4fun [3] was used to predict the abundances of functional genes based on the 16S rRNA amplicon data. The two methods agree well on the more readily identifiable FOAM ortholog groups (Additional file 7: Table S5), e.g. methanogenesis, formaldehyde oxidation (h4mpt pathway), methane oxidation to CO_2_, TCA cycle, hydrocarbon degradation, pyruvate fermentation and homoacetogenesis. We also observed insignificant correlations and even inconsistencies between Tax4fun dataset and metagenomics, notably hydrolysis of polymers, nitro-aromatics degradation, response to oxidative stress and other aromatics degradation. These inconsistencies indicated the relatively poor understanding of genes and taxon involved in some of the considered functions.

Only functional genetic predictions that had significant correlation with the observed metagenomic data were used for further analysis. Methanogenesis, formaldehyde oxidation (H4MPT pathway), methane oxidation, pyruvate fermentation, homoacetogenesis (from acetyl-coA I) and TCA cycle were found to be significantly correlated with PCo1 of the DOM, while hydrocarbon degradation was negatively correlated (Fig. 3e). PCo1 has a cosine value of 0.78 on the first canonical axis of Fig. 5a and explains 44.3% of DOM variance.

**Reference**

1. Menzel P, Ng KL, Krogh A. Fast and sensitive taxonomic classification for metagenomics with Kaiju. Nat Commun. 2016;7:11257.
2. Pinto AJ, Lutgarde R. PCR biases distort bacterial and archaeal community structure in pyrosequencing datasets. PLoS One 2012;7:e43093.
3. Aßhauer KP, Wemheuer B, Daniel R, Meinicke P. Tax4Fun: predicting functional profiles from metagenomic 16S rRNA data. Bioinformatics. 2015;31:2882-4.
