# Supplementary material for: The chemodiversity of paddy soil dissolved organic matter correlates with microbial community at continental scales
Source: Microbiome. 2018 Oct 19;6:187. doi: 10.1186/s40168-018-0561-x (PMC6195703; doi:10.1186/s40168-018-0561-x)
Supplement: Supplementary file 11 — Supplementary methods. (DOCX 32 kb) [file 40168_2018_561_MOESM11_ESM.docx]

**Supplementary methods**

***Soil sampling and characteristics measurements***

The exact sampling sites were selected using a uniform sampling protocol via Google Earth (http://earth.google.com) in Sanjiang, Lianghu and Taihu Plain, whereas the sites in Hani Terrace were determined according to the distribution of large-scale terrace and altitudinal gradient. At each site, a relatively flat area (20 m × 20 m) was identified. Five soil cores (2.5 cm diameter by 15 cm depth) were collected at random within the area, sieved (2 mm) and homogenized to yield one soil sample which was then split into several tubes, sealed and placed in liquid nitrogen for short-distance transportation and in dry ice for long-distance transportation (within 48h). Some tubes were stored at -20℃ or air dried and ground for further physical and chemical analyses. Others were stored in liquid nitrogen for DNA or DOM extraction. We did not analyze replicate samples from individual sampling sites as it was not our goal to assess intra-site variability or determine the chemo-/bio-diversity for each sampling site, but rather to focus on the variability and correlation of the chemo-/bio-diversity across the 88 sites. While longitudinal data is associated with each site, providing intra-site control, these were collected in two different years. However, all data for each analysis were generated on the same sample each time, providing a stable correlative association analysis.

Thawed fresh soils or [air](javascript:void(0);) dried and ground soils were evaluated for physical and chemical properties analyses. Total carbon (TC) and nitrogen (TN) were determined using a Flash 2000 NC Analyzer (Thermo Scientific, MA, USA). The total organic carbon (TOC), which was extracted in the FT-ICR-MS sampler preparing protocol, was measured by an multi NC 3100 TOC analyzer (Analytik Jena AG, Thuringia, Germany) using a high temperature combustion method. pH was measured using the turbid liquid of 5 g soil (dry base) and 10 mL of ultrapure water. Soil moisture was gravimetrically measured by drying the thawed soil at 105℃ for 48 h. Soil total phosphorus (TP) and rapid available phosphorus (Olsen-P) were measured according to standard methods described by Bao [1]. pH and Conductivity were measured in a suspension using 5 g and 10 mL of deionized water [2].

***FT-ICR-MS sample preparation***

DOM was first extracted by adding LC-MS grade water into the soil sample to reach a 1 : 5 (dry-soil : water) mass ratio and then shaking for 2 h in helium. One aliquot of the filtered solution was then adjusted based on DOC concentration (determined by another aliquot) to have a relatively similar concentration and therefore a similar level of bias. Acidified and re-filtered sample (pH = 2) then passed the Bond Elut PPL cartridge (100 mg, 3 ml; Agilent Technologies). These cartridges were previously activated and rinsed using 3 × 3 mL LC-MS grade methanol (sigma-Aldrich) and 3 × 3 mL LC-MS grade water (pH = 2). After rinsing with two cartridge volumes of LC-MS grade water (pH = 2) and drying with ultrapure N_2_ gas, DOM was collected using methanol. Extraction efficiencies (58 ± 20%, 65 ± 14%, 49 ± 18% and 50 ± 17% on average for Hani, Sanjiang, Taihu and Lianghu) were calculated by drying an aliquot of methanol eluate with nitrogen-blow and re-dissolution with ultrapure water. Both acidification and solid phase extraction resulted in loss of part of the soil DOM. Although these procedures narrowed the scope of DOM, the molecular abundances were still comparable. Therefore, the selection of DOM molecules by each procedure was constant. DOC concentration was measured and then an aliquot of eluate was diluted to 40 mg C/L so that the final concentration introduced into the FT-ICR-MS was 20 mg C/L. Before FT-ICR-MS operation, methanol eluates were diluted 1:1 (v/v) with LC-MS grade water and re-filtered with rinsed 0.2 μm PTFE syringe filters. Three blank samples were also processed using equivoluminal LC-MS grade water instead of soil-water suspension during the sample preparing process. Three DOM replicates were prepared for each site and mixed to one samples to mitigated artifacts resulting from the complex sample preparation procedure.

***FT-ICR-MS data analysis***

The evaluation process of FT-ICR-MS data of natural DOM samples has mostly been based on molecular formula assignment, which is extremely challenging. For example, for masses higher than 600 Da, more than 15 different molecular formulae can be calculated for each detected mass within a mass tolerance of 1 ppm and elements restricted to C, H, O, N, P ( ≤ 1) and S ( ≤ 1) [3]. Moreover, most of the automated computer routines did not consider the isotope effect, which together with the uneven distribution of molecules could result in more false annotations. To overcome this problem, different spectral peaks were clustered within a mass tolerance into operational units, similar to the data preparation for amplicon sequencing data.

Multi-point calibration was conducted in DataAnalysis™ 4.2 (Bruker Daltonik GmbH, Bremen, Germany), using “Quadratic” mode with a search range of 0.001 m/z and a custom calibration list including substance of C_23_H_31_O_2_^-^, C_16_H_31_O_2_^-^, C_18_H_35_O_2_^-^, C_24_H_29_O_6_^-^, C_25_H_31_O_8_^-^, C_38_H_75_O_8_^-^. These substances were annotated and were chosen for their higher occurrence frequency. We clustered the spectral peaks from 91 spectra (88 soil DOM spectra and 3 blank spectra) with m/z [difference](javascript:void(0);) ratios less than 1$\times10$^-6^ considered as a cluster (Additional file 1: Fig. S5). In each cluster, the longest distance between two peaks was constrained to less than 1$\times10$^-6^ m/z [difference](javascript:void(0);) ratio. After the one spectrum by one spectrum clustering, we re-clustered the peaks using cluster centers, from large clusters (with more peaks) to small clusters to eliminate the influence of operation order. Spectrum signal intensities were normalized to the sum of all signals (sum-normalized intensity) after removing peaks clustered together with blank peaks and singletons, which were referred as compounds from here on. The average m/z value of the peaks in each cluster was used as the m/z of this compound. Peaks in fewer than 10 samples were not considered to be annotated.

Annotated molecules were assigned to compound categories [4]: polycyclic aromatics; polyphenols and polycyclic aromatics with aliphatic chains (we use “polyphenols” for simplification); phenolics and highly unsaturated compounds (“phenolics” for simplification); unsaturated aliphatics and aromatics with aliphatic chains (“unsaturated aliphatics” for simplification); saturated fatty, sulfonic acids and carbohydrates (“carbohydrates” for simplification); N-containing compounds, i.e. peptides (“peptides” for simplification). Due to the lower recovery efficiency by solid phase extraction and the lower ionization efficiency by FT-ICR-MS for carbohydrates compared to many other component groups, the abundance of carbohydrates (ranged at (0.6-1.0, 1.5-2.3) in van Krevelen diagram) might be underestimated [5]. Therefore, comparisons were only performed between the same categories of different samples.

**References**

1. Bao SD. Agro-chemical analysis of soil. 3rd ed. Beijing: China Agricultural Press; 2000. p. 71-87.
2. Fierer N, Jackson RB. The diversity and biogeography of soil bacterial communities. Proc Natl Acad Sci USA. 2006;103:626–31.
3. Koch BP, Dittmar T, Witt M, Kattner G. Fundamentals of molecular formula assignment to ultrahigh resolution mass data of natural organic matter. Anal Chem. 2007;79:1758–63.
4. Šantltemkiv T, Kai F, Dittmar T, Hansen BM, Thyrhaug R, Nielsen NW, et al. Hailstones: A window into the microbial and chemical inventory of a storm cloud. Plos One. 2013;8:e53550.
5. Seifert AG, Roth VN, Dittmar T, Gleixner G, Breuer L, Houska T, et al. Comparing molecular composition of dissolved organic matter in soil and stream water:Influence of land use and chemical characteristics. Sci Total Environ. 2016;571:142-52.
